# A single relapse induces worsening of disability and health-related quality of life in patients with neuromyelitis optica spectrum disorder

**DOI:** 10.3389/fneur.2023.1099376

**Published:** 2023-04-11

**Authors:** Achim Berthele, Michael Levy, Dean M. Wingerchuk, Sean J. Pittock, Shulian Shang, Adrian Kielhorn, Minying Royston, Guido Sabatella, Jacqueline Palace

**Affiliations:** ^1^Department of Neurology, School of Medicine, Technical University Munich, Klinikum Rechts der Isar, München, Germany; ^2^Massachusetts General Hospital and Harvard Medical School, Mass General Neurology, Boston, MA, United States; ^3^Mayo Clinic, Scottsdale, AZ, United States; ^4^Mayo Clinic, Rochester, MN, United States; ^5^Alexion, AstraZeneca Rare Disease, Boston, MA, United States; ^6^Department of Clinical Neurology, John Radcliffe Hospital, Oxford, United Kingdom

**Keywords:** neuromyelitis optica, relapse, eculizumab, AQP4, HRQoL – health-related quality of life

## Abstract

**Background:**

Cumulative damage from multiple relapses in neuromyelitis optica spectrum disorder (NMOSD) is associated with poor health-related quality of life (HRQoL) and long-term disability in patients positive for anti-aquaporin 4 antibodies (AQP4+). This study assessed the effect of an individual relapse on HRQoL and disability outcomes in AQP4+ NMOSD.

**Methods:**

Post hoc analyses of data pooled from the PREVENT study and its open-label extension, which evaluated the efficacy and safety of eculizumab in AQP4+ NMOSD, examined the effect of a single relapse on 3 disability and 4 HRQoL outcome measures. Assuming the effect of 1 relapse extends to multiple relapses, an extrapolation was done to assess the effect of 2 relapses on these outcomes.

**Results:**

In 27 patients (placebo: *n* = 20; eculizumab: *n* = 7) experiencing an independently adjudicated relapse, 1 relapse led to significantly worse disability (modified Rankin Scale and Expanded Disability Status Scale [EDSS]) and HRQoL (36-item Short-Form Health Survey mental and physical component summaries; European Quality of Life 5-Dimension questionnaire 3-Level visual analogue scale and utility index) scores. In 4 of 7 outcomes, clinically meaningful worsening was more likely for relapsing versus non-relapsing patients (*n* = 116). Extrapolating the effect of 2 relapses predicted that clinically meaningful worsening was more likely in 6 out of 7 outcomes, including EDSS, for patients experiencing multiple relapses versus patients experiencing no relapses.

**Conclusion:**

Findings from these clinical trial data demonstrate that a single NMOSD relapse can worsen disability and HRQoL, underscoring the role of relapse prevention in improving long-term outcomes in patients with AQP4+ NMOSD.

## 1. Introduction

Neuromyelitis optica spectrum disorder (NMOSD) is a rare, disabling, autoimmune disease of the central nervous system, characterized by relapses (attacks) that primarily target the optic nerve (optic neuritis) and spinal cord (myelitis) (1, 2). In NMOSD patients positive for anti-aquaporin 4 antibodies (AQP4+), inflammatory attacks depend on complement activation (1, 3). In the majority of patients with NMOSD, the risk of relapses is enduring, with up to 90% of patients with AQP4+ NMOSD experiencing relapses during the course of their disease (4, 5).

Relapse severity in NMOSD is unpredictable, leading to varying degrees of neurological dysfunction (4, 6). Recovery from these relapses is often poor, driving a stepwise accumulation of neurological disability (2, 7). Consequently, a substantial proportion of patients with NMOSD are unable to walk without unilateral or bilateral assistance within several years of disease onset (2, 5, 7–9). In addition, patients with NMOSD commonly report poor health-related quality of life (HRQoL) because of pain and physical disability, which can limit the performance of usual activities (e.g., work, social engagements, daily tasks) (10–12). However, the effect of a single NMOSD relapse on patients’ disability or HRQoL has not been previously reported in the literature. Although acute treatments, such as high-dose steroids or plasma exchange, may be administered to improve short-term outcomes following a relapse, more studies are needed to provide insights on the effect, and the durability of the effect, of a single NMOSD relapse on clinical measures of disability and HRQoL. Such studies may help to inform treatment decisions aimed at relapse prevention.

## 2. Methods

### 2.1. Study design

Post hoc analyses were conducted using pooled data from the pivotal, phase 3 PREVENT (Prevention of Relapses in Neuromyelitis Optica) study (NCT01892345) and its open-label extension (OLE). The phase 3 PREVENT study was a randomized, double-blind, placebo-controlled, time-to-event trial evaluating the efficacy and safety of eculizumab in patients with AQP4+ NMOSD (13). Patients at 70 sites across 18 countries were enrolled from April 1, 2014 to October 31, 2017. At enrollment, patients were randomly assigned at a 2:1 ratio to receive eculizumab or placebo based on results of the Expanded Disability Status Scale (EDSS) and the use of concomitant immunosuppressive therapy; all relapses were adjudicated by an independent panel of investigators. Patients who completed the phase 3 PREVENT study could enter the OLE and receive treatment with eculizumab (13).

In this study, post hoc analyses were conducted using data from the first relapse of patients and non-relapsing patients (those who did not experience any adjudicated relapses) through the OLE interim cut-off date (July 31, 2019). Among relapsing patients, only data from the first adjudicated relapse were included in this analysis (13).

### 2.2. Standard protocol approvals, registrations, and patient consents

The PREVENT trial was conducted in accordance with the principles of the Declaration of Helsinki, (14) the International Conference on Harmonisation guidelines for Good Clinical Practice, (15) and applicable regulatory requirements. Alexion Pharmaceuticals (now Alexion, AstraZeneca Rare Disease) designed the trial in consultation with 2 academic authors, provided the trial agents, and performed data analysis. Confidentiality agreements were in place between the authors and Alexion Pharmaceuticals.

This study reports data from a post hoc analysis of PREVENT and its OLE, which were approved by institutional review boards of participating institutions. All study participants provided written informed consent.

### 2.3. Patient population

The phase 3 PREVENT study included adults (aged ≥ 18 years) with AQP4+ neuromyelitis optica (NMO) or NMOSD (according to 2006 and 2007 criteria) (13). Patients were required to have 2 or more relapses in the past year or 3 or more relapses in the past 2 years (with at least 1 relapse in the past year) and an EDSS score ≤ 7.0 (13). Patients from the PREVENT study were mostly women (91%); had moderate to severe disability based on EDSS (median score: 4.0, range: 1.0–7.0), modified Rankin Scale (mRS) (median score: 2.0, range: 0–4), and Hauser Ambulation Index (HAI) (median score: 2.0, range: 0–8) disability scores; and a mean annualized relapse rate at baseline of 1.99 (±0.94) during the previous 24 months (13). The baseline characteristics of these patients were well balanced between those who received eculizumab versus those who received placebo (13). In addition, 32% of patients previously received rituximab, but patients were not eligible if rituximab was administered within 3 months before screening. Concomitant immunosuppressive therapy was administered at a stable dosing regimen to 76% of patients during the trial (13).

### 2.4. Outcomes

In the PREVENT study, disability was assessed using 3 physician-recorded instruments: the EDSS (neurological disability) ranging from 0 to 10, (16) the HAI (mobility) ranging from 0 to 9, (17) and the mRS (activity) ranging from 0 to 6 (18, 19). Higher scores for the EDSS, HAI, and mRS indicate increased disability. Patient-reported HRQoL was assessed using the European Quality of Life 5-Dimension questionnaire 3-Level (EQ-5D-3L) visual analogue scale (VAS) ranging from 0 to 100, the EQ-5D-3L index score ranging from 0 to 1, and the physical component summary (PCS) score and mental component summary (MCS) scores of the 36-Item Short-Form Health Survey (SF-36), both ranging from 0 to 100 (20). Higher scores for the EQ-5D-3L VAS, EQ-5D-3L index score, SF-36 PCS, and SF-36 MCS indicate better health and reduced disability.

### 2.5. Statistical analysis

In these post hoc analyses, 3 analytical strategies were employed. The first analysis used data from relapsing patients to calculate the mean change from pre-relapse (defined by the last assessment of each instrument prior to a relapse) to 30, 90, and 120 days post-relapse for all disability and HRQoL outcomes. Comparisons between pre-relapse and post-relapse scores were conducted using a Wilcoxon signed rank test. Statistical significance was defined as *p* < 0.05.

For the second analysis, clinically meaningful worsening after a relapse was evaluated for each outcome based on the proportion of patients who experienced worsening from pre-relapse to 30, 90, and 120 days post-relapse. Clinically meaningful worsening implies the smallest worsening of symptoms to influence a physician’s management care plan for a patient. For each instrument, clinically meaningful worsening was defined as follows: EDSS: ≥2.0-point increase if baseline score = 0, ≥1.0-point increase if baseline score ≥1.0–5.0, ≥0.5-point increase if baseline score ≥5.5; (21, 22) HAI: ≥2.0-point increase if baseline score = 0, ≥1.0-point increase if baseline score ≥1; (17) mRS: 1.0-point increase; (23) EQ-5D-3L VAS: 8.0-point decrease; (24) EQ-5D-3L Index: 0.18-point decrease; (25) SF-36 PCS and MCS: 5.0-point decrease for each (26, 27).

The third analysis examined the probability of experiencing clinically meaningful worsening between relapsing and non-relapsing patients using a logistic regression model that adjusted for the baseline score of each instrument. For all patients, baseline scores were determined at the beginning of the phase 3 PREVENT study. The model used the last score of each instrument as follows: for those who did not roll over to the OLE, the last score before the end of the phase 3 PREVENT study was used; for those who entered the OLE, the last score before the OLE interim cut-off date (as of July 31, 2019) was used.

In addition to the analytical approaches described above, a logistic regression model was used to estimate the effect of multiple relapses on disability and HRQoL outcomes. Using baseline values for each of the 7 instruments administered during the phase 3 PREVENT study as a starting point, (13) the effect of 2 relapses was estimated by doubling the effect of the first relapse on each instrument and comparing the likelihood of clinically meaningful worsening between patients with 2 relapses versus non-relapsing patients.

The clinical characteristics of relapsing patients were summarized using descriptive statistics (*n*, %). All analyses were conducted using SAS version 9.4.

## 3. Results

### 3.1. Patient characteristics

Data from 27 patients with an adjudicated relapse recorded during the phase 3 PREVENT study or OLE were evaluated, of which 20 relapses occurred in placebo-treated patients. The clinical characteristics of relapsing patients are shown in Table 1. Most on-study relapses were diagnosed as myelitis (70.4%). Twenty-six patients (96.3%) received acute treatments for the relapse. One patient was lost to follow-up at 30, 90, and 120 days post-relapse for all outcomes except the EDSS and HAI.

**Table 1:** Clinical characteristics of relapsing patients.

	PREVENT (*n* = 23)		OLE (*n* = 4)	Total (*N* = 27)
	Placebo (*n* = 20)	Eculizumab (*n* = 3)	Eculizumab^a^	Total
Adjudicated relapse type, *n* (%)				
Optic neuritis	2 (10.0)	2 (66.7)	3 (75.0)	7 (25.9)
Myelitis	17 (85.0)	1 (33.3)	1 (25.0)	19 (70.4)
Other	1 (5.0)	0 (0.0)	0 (0.0)	1 (3.7)
Acute treatment post-relapse, *n* (%)				
Plasma exchange only	2 (10.0)	0 (0.0)	0 (0.0)	2 (7.4)
High-dose steroid only^b^	13 (65.0)	1 (33.3)	2 (50.0)	16 (59.3)
IVIG only	0 (0.0)	0 (0.0)	0 (0.0)	0 (0.0)
Both plasma exchange and high-dose steroid^b^	4 (20.0)	2 (66.7)	2 (50.0)	8 (29.6)
No acute treatment	1 (5.0)	0 (0.0)	0 (0.0)	1 (3.7)

^a^All patients in the OLE were treated with eculizumab. Five patients in the OLE had 1 or more adjudicated relapses; however, 1 patient had a first relapse during the PREVENT study and was therefore counted in the PREVENT pool. ^b^Includes both high-dose oral steroids and IV methylprednisolone. IV, intravenous; IVIG, intravenous immunoglobulin; OLE, open-label extension; PREVENT, Prevention of Relapses in Neuromyelitis Optica.

### 3.2. Disability and HRQoL following a single relapse

**Figure 1:**
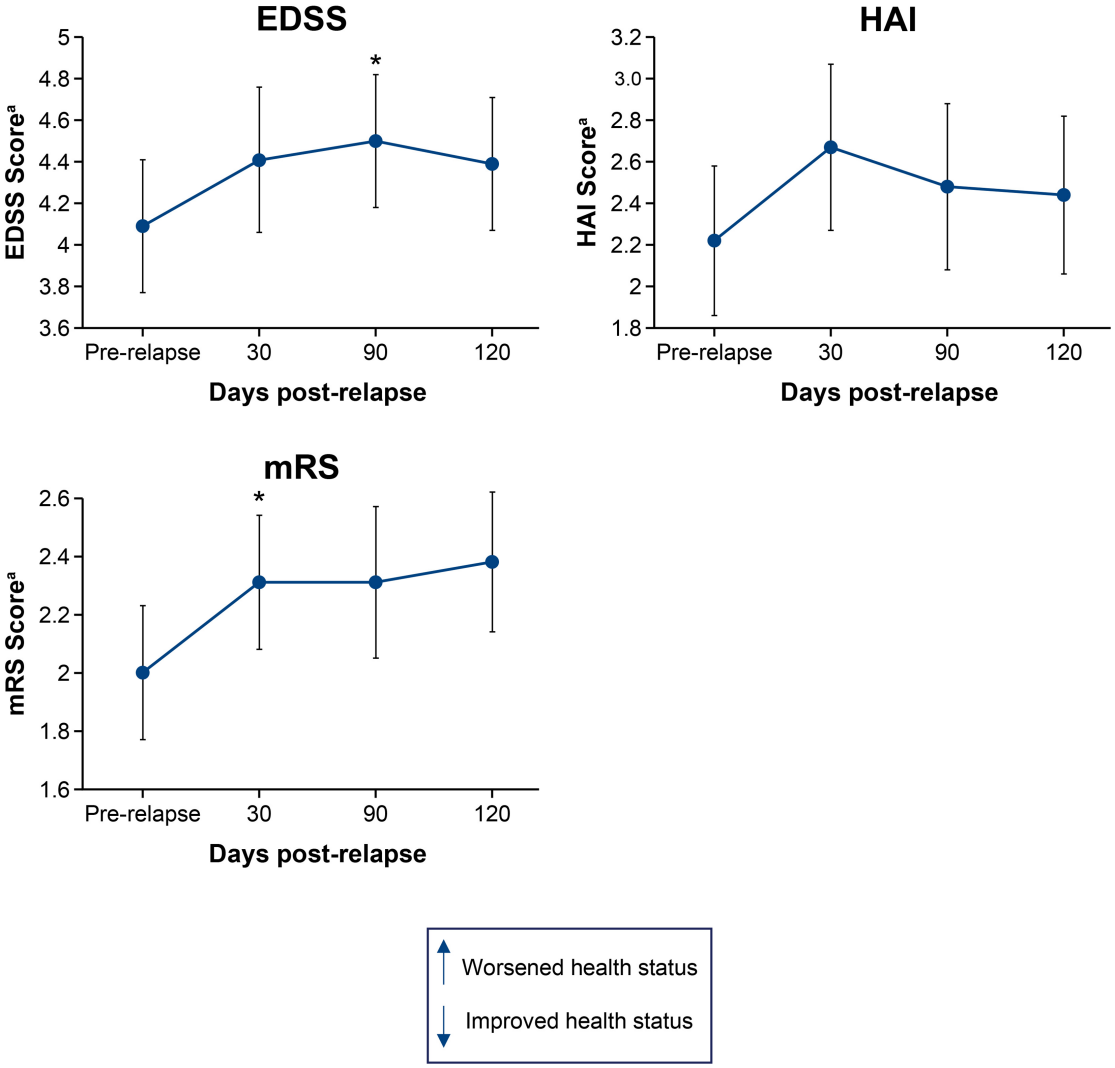
Mean change in EDSS, HAI, and mRS scores from pre-relapse to 30, 90, and 120 days post-relapse. *p < 0.05 for the mean change from pre-relapse to post-relapse. ^a^Higher scores indicate worsened health status. EDSS, Expanded Disability Status Scale; HAI, Hauser Ambulation Index; mRS, modified Rankin scale.

**Figure 2:**
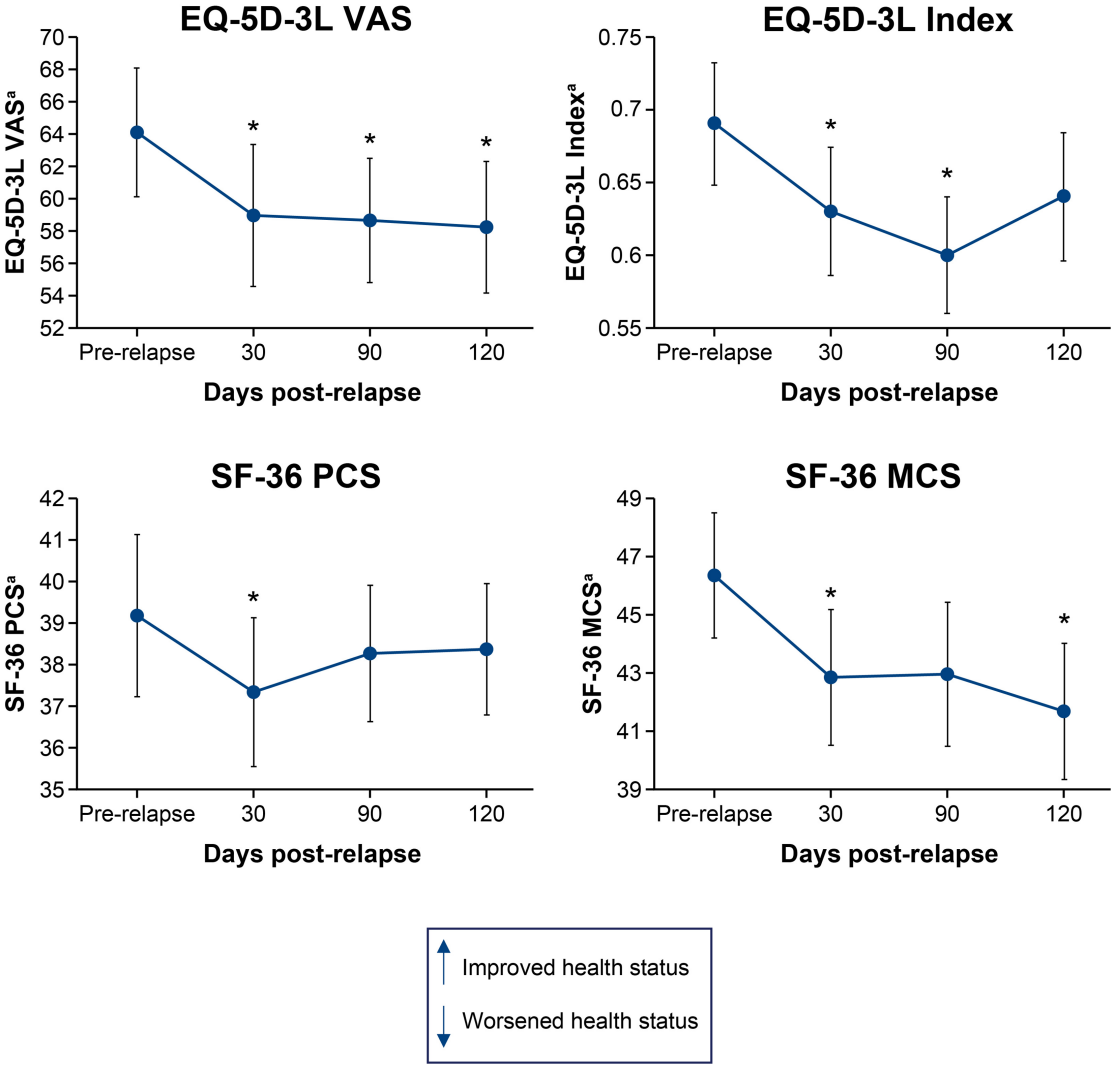
Mean change in EQ-5D-3L VAS, EQ-5D-3L Index, SF-36 PCS, and SF-36 MCS scores from pre-relapse to 30, 90, and 120 days post-relapse. *p < 0.05 for the mean change from pre-relapse to post-relapse. ^a^Lower scores indicate worsened health status. EQ-5D-3L, European Quality of Life 5-Dimension questionnaire 3-Level; HRQoL, health-related quality of life; MCS, mental component summary; PCS, physical component summary; SF-36, 36-Item Short-Form Health Survey; VAS, visual analogue scale.

Figures 1, 2 show the mean change in disability and HRQoL scores over time in relapsing patients. Across all endpoints, the effect of a single relapse was noticeable and permanent. Mean scores in disability were worse (*p* < 0.05) after the relapse than before the relapse for 1 of the physician-recorded instruments assessed at 30 days (mRS, *p* = 0.035) and 1 assessed at 90 days (EDSS, *p* = 0.027). Mean HRQoL scores were worse (*p* < 0.05) after the relapse than before the relapse in all 4 instruments assessed at 30 days (EQ-5D-3L VAS, *p* = 0.012; EQ-5D-3L Index, *p* = 0.005; SF-36 PCS, *p* = 0.018; SF-36 MCS, *p* = 0.013), 2 of the instruments assessed at 90 days (EQ-5D-3L VAS, *p* = 0.047; EQ-5D-3L Index, *p* = 0.011), and 2 of the instruments assessed at 120 days (EQ-5D-3L VAS, *p* = 0.040; SF-36 MCS, *p* = 0.013). Of note, mean scores on the EQ-5D-3L VAS were worse (*p* < 0.05) at all time points post-relapse.

### 3.3. Visual acuity change in patients with myelitis after a single relapse

A total of 19/27 patients were determined to have myelitis by independent adjudication. Using the Kurtzke functional systems scores, the mean pre-relapse visual acuity for patients with myelitis was 2.4 (standard deviation [SD]: 2.14) (16). The mean change in visual acuity did not vary considerably post-relapse after 30 days (−0.1 [SD: 0.32]), 90 days (−0.1 [SD: 0.33]), or 120 days (0.1 [SD: 0.57]), indicating that visual acuity is not likely to have negatively impacted disability and HRQoL outcomes for patients with myelitis.

### 3.4. Clinically meaningful worsening after a single relapse

**Figure 3:**
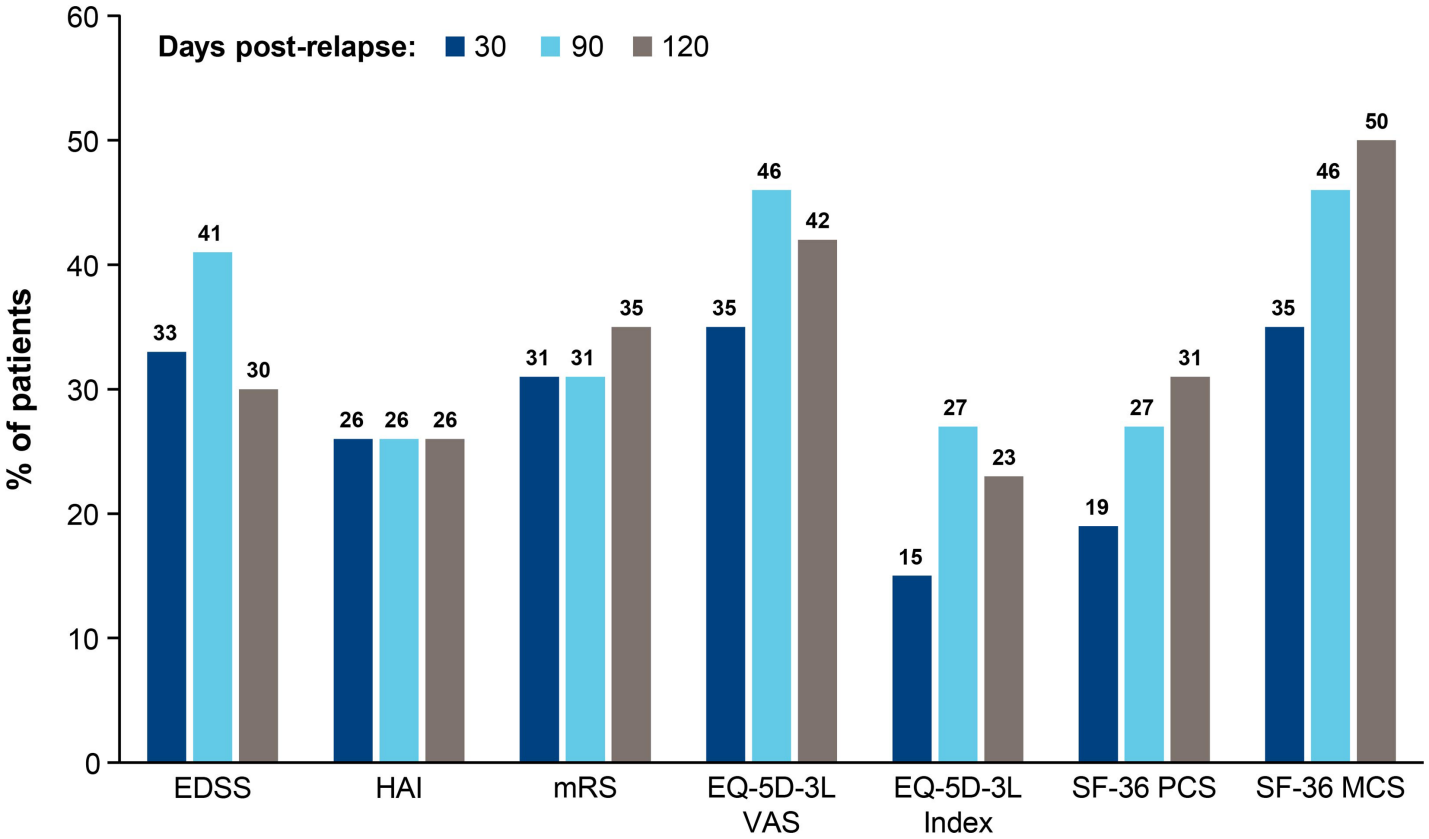
Proportion of patients who experienced clinically meaningful worsening^a^ between pre- and post-relapse scores at 30, 90, and 120 days. ^a^Clinically meaningful worsening implies the smallest worsening of symptoms to impact the management care plan of a patient. For each instrument, clinically meaningful worsening was defined as follows: EDSS: ≥ 2.0-point increase if baseline score = 0, ≥ 1.0-point increase if baseline score ≥ 1.0–5.0, ≥ 0.5-point increase if baseline score ≥ 5.5; HAI: ≥ 2.0-point increase if baseline score = 0, ≥ 1.0-point increase if baseline score ≥ 1; mRS: 1.0-point increase; EQ-5D-3L VAS: 8.0-point decrease; EQ-5D-3L Index: 0.18-point decrease; SF-36 PCS: 5.0-point decrease; SF-36 MCS: 5.0-point decrease. EDSS, Expanded Disability Status Scale; EQ-5D-3L, European Quality of Life 5-Dimension questionnaire 3-Level; HAI, Hauser Ambulation Index; MCS, mental component summary; mRS, modified Rankin scale; PCS, physical component summary; SF-36, 36-Item Short-Form Health Survey; VAS, visual analogue scale.

An alternative approach to quantify the effect of a single relapse is to measure the proportion of patients exceeding a minimum degree of worsening. For this analysis, the published minimal clinically important difference thresholds were used. Results in Figure 3 show that 15%–50% of relapsing patients experienced clinically meaningful worsening of disability and HRQoL after a single relapse. Between 30 and 90 days post-relapse, the proportion of patients with clinically meaningful worsening increased for 5 of the instruments (EDSS, EQ-5D-3L VAS, EQ-5D-3L Index, SF-36 PCS, and SF-36 MCS) and was stable for 2 of the instruments (HAI and mRS). Between 90 and 120 days post-relapse, the proportion of patients who experienced clinically meaningful worsening decreased by 11% for the EDSS and was similar for all other domains, suggesting a stabilization of relapse symptoms.

**Figure 4:**
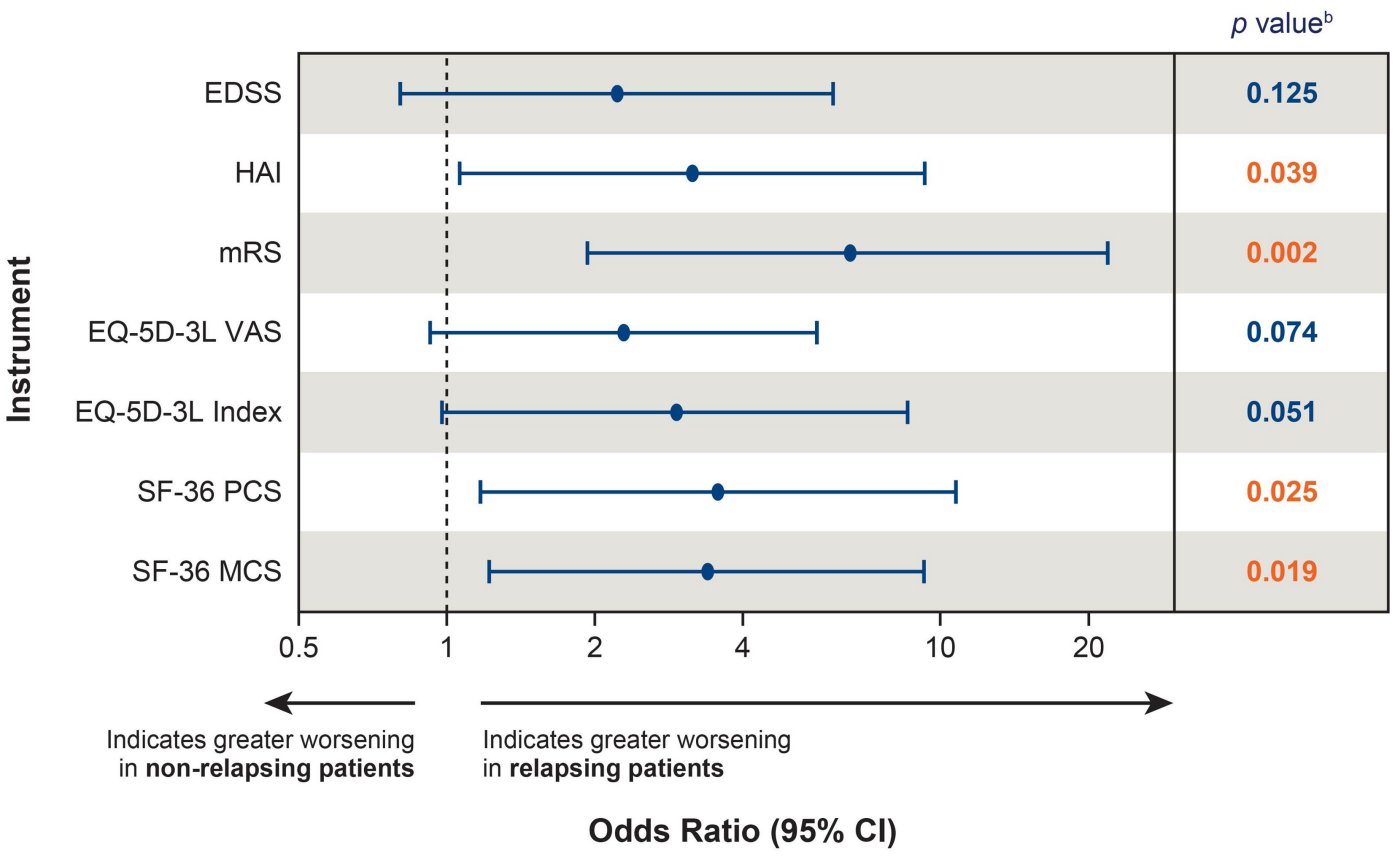
Likelihood of clinically meaningful worsening^a^ between relapsing (*n* = 27) and non-relapsing (*n* = 116) patients. ^a^Clinically meaningful worsening was calculated between the PREVENT baseline score and the score at the end of the OLE; for patients who did not roll over to the OLE, the score at the end of the PREVENT study was used. ^b^Red font indicates *p* <  0.05. CI, confidence interval; EDSS, Expanded Disability Status Scale; EQ-5D-3L, European Quality of Life 5-Dimension questionnaire 3-level; HAI, Hauser Ambulation Index; MCS, mental component summary; mRS, modified Rankin scale; OLE, open-label extension; PCS, physical component summary; PREVENT, Prevention of Relapses in Neuromyelitis Optica; SF-36, 36-Item Short-Form Health Survey; VAS, visual analogue scale.

The effect of a single relapse on disability and HRQoL can also be investigated by comparing the likelihood of worsening between relapsing (*n* = 27) and non-relapsing (*n* = 116) patients. When comparing the likelihood of clinically meaningful worsening between relapsing and non-relapsing patients during the follow-up period (mean: 2.9 years for both groups; median: 2.99 years and 2.94 years, respectively), relapsing patients were more likely to experience clinically meaningful worsening in 4 of the 7 instruments (HAI, mRS, SF-36 PCS, and SF-36 MCS) than non-relapsing patients (Figure 4), indicating that a single relapse drives not only increased disability, but also poor HRQoL in patients with NMOSD.

### 3.5. Estimating the effect of multiple relapses on disability and HRQoL

**Figure 5:**
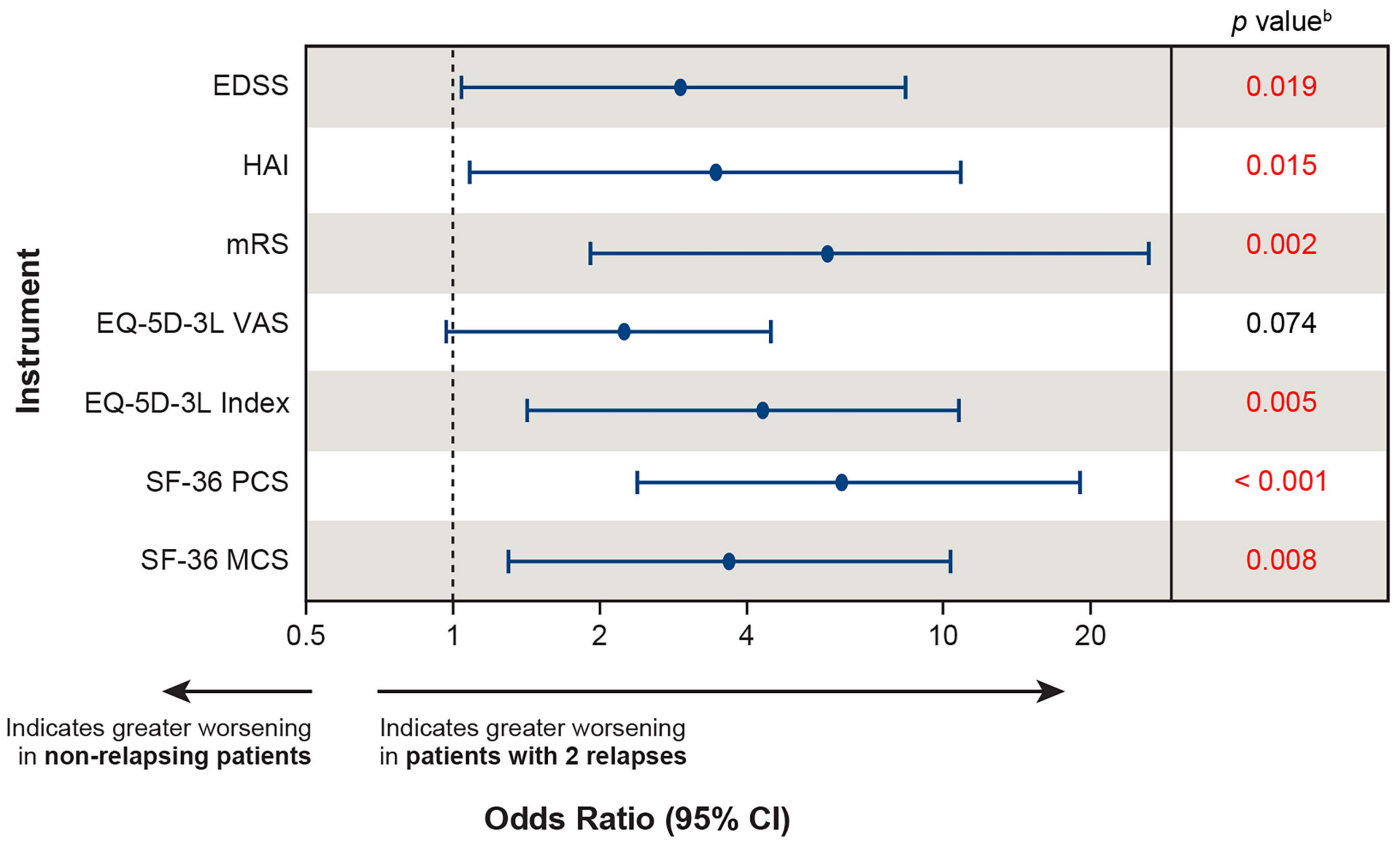
Extrapolating^a^ the likelihood of clinically meaningful worsening in patients experiencing 2 relapses versus non-relapsing patients. ^a^The effect of 2 relapses was estimated by doubling the effect of the first relapse on each instrument using baseline values from the PREVENT study and calculating the likelihood of clinically meaningful worsening between patients with 2 relapses versus non-relapsing patients. ^b^Red font indicates *p* <  0.05. CI, confidence interval; EDSS, Expanded Disability Status Scale; EQ-5D-3L, European Quality of Life 5-Dimension questionnaire 3-level; HAI, Hauser Ambulation Index; MCS, mental component summary; mRS, modified Rankin scale; OLE, open-label extension; PCS, physical component summary; PREVENT, Prevention of Relapses in Neuromyelitis Optica; SF-36, 36-Item Short-Form Health Survey; VAS, visual analogue scale.

All recent pivotal trials in patients with AQP4+ NMOSD used time to first relapse as the primary endpoint. Thus, unfortunately, no efficacy data are available to measure the benefit of reducing multiple relapses in a disorder where, despite treatment with immunosuppressant therapies, patients experience a median number of 5 relapses over the course of the disease (median annual rates: optic neuritis: 0.38, myelitis: 0.53) (4). To highlight just how damaging multiple relapses are for patients, we extrapolated the effect of 2 relapses by doubling the effect of a single relapse on disability and HRQoL outcomes using data from the phase 3 PREVENT study. We assumed that, across all patients, the effect of a second relapse would lead to changes in each of the disability and HRQoL instruments that were identical to those of the first relapse. A logistic regression analysis was conducted comparing the likelihood of clinically meaningful worsening between patients with 2 relapses versus those with no relapses. Patients with 2 relapses were more likely to experience clinically meaningful worsening in 6 out of the 7 instruments (EDSS, HAI, mRS, EQ-5D-3L index, SF-36 PCS, and SF-36 MCS) compared with non-relapsing patients (Figure 5), underscoring the severity of the damage that can occur from multiple relapses.

## 4. Discussion

Previous evidence has established relapses as a primary driver of disability accumulation in patients with NMOSD by comparing outcomes between relapsing and monophasic patients (those who experienced only a single attack at disease onset) (2). Our results add to this literature by showing that a single relapse induces worsening of disability, specifically in patients with AQP4+ NMOSD who exhibit a relapsing disease course, by comparing outcomes immediately before and up to 120 days after a relapse; these findings are consistent with recently published literature (28). Notably, in our study, data from multiple physician- and patient-reported instruments demonstrated that patients who experienced a single relapse had significant and sustained clinically meaningful worsening, not only of disability, but also of HRQoL outcomes.

Adding to the evidence that patients with NMOSD have reduced HRQoL compared with the general population, (11, 12) our results identify a single relapse as a key factor that diminishes HRQoL in patients with AQP4+ NMOSD. This finding aligns with the results of a recent analysis that showed HRQoL significantly correlated with the number of relapses experienced by patients with NMOSD, with a greater number of relapses leading to poorer HRQoL (11). These findings are in agreement with a report from 2007 that showed a definitive secondary progressive disease course (usually progressive myelopathy) is very uncommon in patients with NMOSD, (29) and limited evidence on the existence of secondary progressive disease in NMOSD has been published since then (30). Furthermore, the results of our logistic regression model show that patients who experienced a single relapse are more likely to have clinically meaningful worsening of disability and HRQoL outcomes than non-relapsing patients.

Extrapolating the likelihood of clinically meaningful worsening of disability and HRQoL outcomes after 2 relapses using baseline data from the phase 3 PREVENT study provides a wider perspective on the importance of relapse reduction. The benefits of drugs that have shown to reduce the risk and burden associated with a single relapse in clinical trials may not be fully appreciated by stakeholders unfamiliar with NMOSD (e.g., fund holders and payers). For instance, the benefit of a risk reduction of 94% as shown for eculizumab in the phase 3 PREVENT study, projected over several years, could prevent patients from experiencing any relapse-caused worsening of neurological disability or HRQoL. This would allow patients to maintain their levels of health relative to NMOSD at the start of treatment. Therefore, relapse prevention should be the main goal of treatment in patients with NMOSD (7, 8, 28, 31).

It is also important to note that only a fraction of patients exhibited clinically meaningful worsening on the 7 instruments assessed in our study (Figure 3). These results suggest that quick detection of NMOSD relapses and appropriate selection of acute interventions can limit the occurrence of clinically meaningful, sustained worsening. However, further research is needed to determine which acute treatments offer the best outcomes, highlighting the importance of using therapies that prevent relapses before they ever occur. Furthermore, it is likely that the results of this study have underestimated the effect of multiple relapses on patient outcomes. Our analyses included data from patients who were treated with eculizumab, which, based on physician ranking in the phase 3 PREVENT study, appeared to promote less severe relapses when compared with placebo.

### 4.1. Limitations

First, the sample size for relapsing patients was small (*n* = 27), which could limit the precision of the estimates of change associated with a single relapse from our findings. For the extrapolation analysis, it was assumed that patients would experience a second relapse of the same severity, meaning that all scores on the 7 instruments would change linearly after a second relapse. This assumption may not be accurate for all patients because relapse severity and recovery following a relapse are unpredictable and accurately quantifying the severity of a relapse or recovery following a relapse in patients who have already had a relapse is challenging due to a lack of multiple relapses observed in a clinical trial. In addition, as assessments were predetermined by the clinical trial schedule, the timing of the last available assessment prior to a relapse varied across patients; relapses could have occurred at any time. Due to the small sample size, neither the severity of the effect nor the relapse type could be considered in this analysis. This also applies to the effect of prior relapses, or the acute treatment given during the relapse. The effect of relapses on vision loss also could not be incorporated in this analysis due to the low number of patients with optic neuritis. Therefore, the clinical effect of relapses on the vision of patients and the resulting HRQoL are likely to be underreported. Cognitive or psychological factors, such as depression or anxiety, were also not specifically investigated, which could have contributed to the HRQoL outcomes reported in this study (32). Lastly, per inclusion criteria for the phase 3 PREVENT study, this analysis evaluated patients with a moderate–severe baseline disability derived from at least 2 relapses in the 2–3 years prior to enrollment. Thus, it is possible that the impact of a single relapse in patients with a less aggressive disease course might be different and would be better investigated with a dedicated study.

## 5. Conclusion

In this post hoc analysis of clinical trial data, patients with AQP4+ NMOSD who experienced a single relapse exhibited a sustained worsening of disability and HRQoL outcomes over time. These results further confirm that disability in NMOSD is driven by relapses. By analyzing data from the independently adjudicated, placebo-controlled, global PREVENT clinical trial and its OLE using multiple physician- and patient-reported instruments, these findings demonstrate the significant role that a single relapse plays in determining disability accumulation in NMOSD. They also highlight that preventing relapses is key to improving long-term outcomes in patients with AQP4+ NMOSD.

## Data availability statement

The datasets presented in this article are not readily available because of ethical and privacy restrictions. Requests to access the datasets should be directed to the corresponding author/s.

## Ethics statement

The studies involving human participants were reviewed and approved by Alexion, AstraZeneca Rare Disease. The patients/participants provided their written informed consent to participate in this study.

## Author contributions

AB, ML, DW, SP, SS, AK, MR, GS, and JP contributed to the conception and design of the study and writing and editing the manuscript. AK acquired the data. SS performed the statistical analysis. All authors contributed to the article and approved the submitted version.

## Funding

The authors declare that this study received funding from Alexion, AstraZeneca Rare Disease, Boston, MA, United States.

## Conflict of interest

AB’s institution received compensations from Alexion, AstraZeneca Rare Disease for participation in the PREVENT study. Outside the submitted work, he has participated in consultancy, speaker bureaus, and advisory boards with Alexion, AstraZeneca Rare Disease, Bayer Healthcare, Biogen, Celgene, Merck Serono, Novartis Pharmaceuticals, Roche, Sanofi Genzyme, and Teva Pharmaceuticals. ML received consulting fees from all 3 manufacturers involved in neuromyelitis optica spectrum disorder clinical trials including Alexion, AstraZeneca Rare Disease, Viela Bio, and Genentech/Roche/Chugai. In addition, he has received consulting fees from Quest Diagnostics, Mitsubishi, and UCB Pharmaceuticals. DW is an employee of the Mayo Clinic and participated on a data safety monitoring or advisory board for Roche, Viela Bio, Genentech, Biogen, Reistone, TG Therapeutics, Celgene, and Novartis, with fees paid directly to himself; and for Alexion, AstraZeneca Rare Disease, with fees paid to his institution. He received grants for clinical trials through Alexion, AstraZeneca Rare Disease and TerumoBCT paid directly to his institution and was personally paid consulting fees by Mitsubishi Tanabe. SP and his institution have received honorariums and travel expenses for attending NMOSD advisory board meetings (MedImmune, Alexion, AstraZeneca Rare Disease), consulting fees (Alexion, AstraZeneca Rare Disease, Euroimmun, Grifols, MedImmune), grant/research support (Alexion, AstraZeneca Rare Disease, the Autoimmune Encephalitis Alliance, Euroimmun, Grifols, MedImmune), and other fees received from the National Institutes of Health and the Guthy-Jackson Charitable Foundation. He has a patent, Patent# 8889102 (Application# 12-678350, Neuromyelitis optica autoantibodies as a marker for neoplasia), issued; another patent, Patent# 9891219B2 (Application# 12-573942, Methods for treating neuromyelitis optica (NMO) by administration of eculizumab to an individual that is aquaporin-4 (AQP4)-IgG autoantibody positive), issued. SS, AK, and GS are employees and stockholders of Alexion, AstraZeneca Rare Disease. MR was an employee and stockholder of Alexion, AstraZeneca Rare Disease during the development of this manuscript. JP has received support for scientific meetings and honorariums for advisory work from Merck Serono, Novartis, Chugai, Alexion, Roche, MedImmune, Argenx, UCB, Mitsubishi, Amplo, Janssen, Sanofi, and grants from Alexion, Roche, MedImmune, and Amplo Biotechnology; has patent ref. P37347WO, a license agreement with Numares for multi-marker MS diagnostics, and ISA shares in AstraZeneca; and acknowledges partial funding by Highly specialized services, NHS, England. The authors declare that this study received funding from Alexion, AstraZeneca Rare Disease, Boston, MA, USA. The funder had the following involvement with the study: study design, collection, analysis, interpretation of data, the writing of this article, the decision to submit it for publication.

## Publisher’s note

All claims expressed in this article are solely those of the authors and do not necessarily represent those of their affiliated organizations, or those of the publisher, the editors and the reviewers. Any product that may be evaluated in this article, or claim that may be made by its manufacturer, is not guaranteed or endorsed by the publisher.
